# Deep learning based ultrasound analysis facilitates precise distinction between parotid pleomorphic adenoma and Warthin tumor

**DOI:** 10.3389/fonc.2024.1337631

**Published:** 2024-02-27

**Authors:** Xi-hui Liu, Yi-yi Miao, Lang Qian, Zhao-ting Shi, Yu Wang, Jiong-long Su, Cai Chang, Jia-ying Chen, Jian-gang Chen, Jia-wei Li

**Affiliations:** ^1^ Department of Medical Ultrasound, Fudan University Shanghai Cancer Center, Shanghai, China; ^2^ Department of Oncology, Shanghai Medical College, Fudan University, Shanghai, China; ^3^ School of AI and Advanced Computing, XJTLU Entrepreneur College (Taicang), Xi’an Jiaotong-Liverpool University, Suzhou, China; ^4^ Department of Oral Pathology, Shanghai Ninth People’s Hospital, Shanghai Jiaotong University, Shanghai, China; ^5^ Department of Neck Surgery, Fudan University Shanghai Cancer Center, Shanghai, China; ^6^ Shanghai Key Laboratory of Multidimensional Information Processing, East China Normal University, Shanghai, China

**Keywords:** deep learning, pleomorphic adenoma, Warthin tumor, ultrasound, diagnosis

## Abstract

**Background:**

Pleomorphic adenoma (PA), often with the benign-like imaging appearances similar to Warthin tumor (WT), however, is a potentially malignant tumor with a high recurrence rate. It is worse that pathological fine-needle aspiration cytology (FNAC) is difficult to distinguish PA and WT for inexperienced pathologists. This study employed deep learning (DL) technology, which effectively utilized ultrasound images, to provide a reliable approach for discriminating PA from WT.

**Methods:**

488 surgically confirmed patients, including 266 with PA and 222 with WT, were enrolled in this study. Two experienced ultrasound physicians independently evaluated all images to differentiate between PA and WT. The diagnostic performance of preoperative FNAC was also evaluated. During the DL study, all ultrasound images were randomly divided into training (70%), validation (20%), and test (10%) sets. Furthermore, ultrasound images that could not be diagnosed by FNAC were also randomly allocated to training (60%), validation (20%), and test (20%) sets. Five DL models were developed to classify ultrasound images as PA or WT. The robustness of these models was assessed using five-fold cross-validation. The Gradient-weighted Class Activation Mapping (Grad-CAM) technique was employed to visualize the region of interest in the DL models.

**Results:**

In Grad-CAM analysis, the DL models accurately identified the mass as the region of interest. The area under the receiver operating characteristic curve (AUROC) of the two ultrasound physicians were 0.351 and 0.598, and FNAC achieved an AUROC of only 0.721. Meanwhile, for DL models, the AUROC value for discriminating between PA and WT in the test set was from 0.828 to 0.908. ResNet50 demonstrated the optimal performance with an AUROC of 0.908, an accuracy of 0.833, a sensitivity of 0.736, and a specificity of 0.904. In the test set of cases that FNAC failed to provide a diagnosis, DenseNet121 demonstrated the optimal performance with an AUROC of 0.897, an accuracy of 0.806, a sensitivity of 0.789, and a specificity of 0.824.

**Conclusion:**

For the discrimination of PA and WT, DL models are superior to ultrasound and FNAC, thereby facilitating surgeons in making informed decisions regarding the most appropriate surgical approach.

## Introduction

Parotid gland is the largest of the three major salivary glands and is also the most common site for the occurrence of salivary gland tumors. The conventional belief is that the majority of primary parotid tumors (75% - 85%) are benign, with pleomorphic adenoma (PA) and Warthin tumor (WT) accounting for up to 93% of benign parotid tumors (1–4). However, PA is not so innocent and harmless because carcinoma ex pleomorphic adenoma (CXPA), a malignant salivary gland tumor typically found in the parotid gland, arises from the “benign” tumor PA (5–7). Early on, this tumor appears benign. Hence, more attention should be paid to the accurate recognition of PA. In contrast, WT is less invasive in nature with rare malignant transformation. However, the differentiation of PA is challenging because both PA and WT present as painless and slow-growing masses (8) and share similar imaging features (9). Even worse, the accuracy of fine needle aspiration cytology (FNAC) in identifying the pathological types of PA and WT is relatively low as reported in the literature (10).

As a result of the difference in biological characteristics between PA and WT, the treatment approaches for PA and WT are fundamentally different. Typically, PA adopts extensive or partial parotidectomy. In contrast, the current trend in managing WT is to opt for extracapsular dissection, and even active surveillance is considered (11–13). Therefore, precise differentiation between PA and WT prior to the surgery is essential in tailoring treatment decisions.

**Figure 1:**
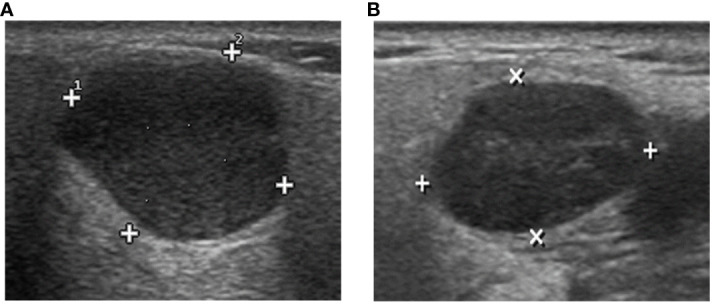
The DL algorithm correctly classified **(A)** biopsy-PA nodule with a WT appearance as PA and **(B)** biopsy-WT nodule with a PA appearance as WT. However, the sonographic features demonstrate similarities in the images, making it difficult to classify the two tumors by ultrasound physicians.

**Figure 2:**
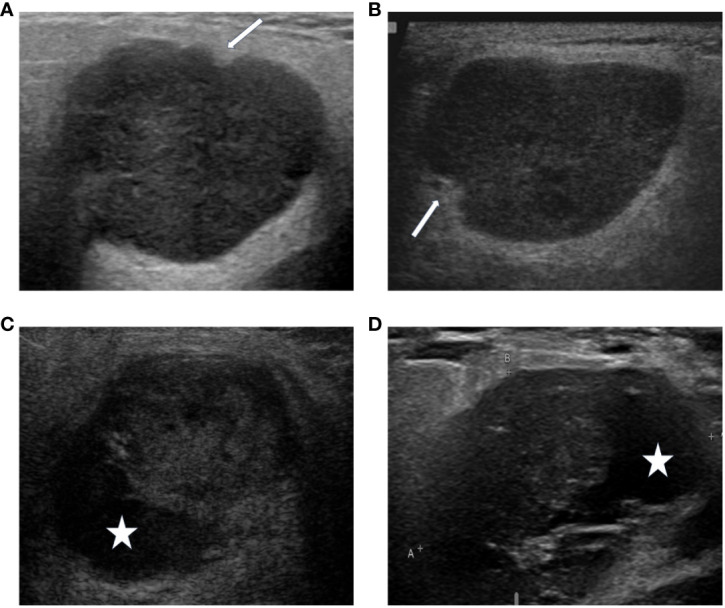
The DL algorithm correctly classified **(A)** biopsy-PA nodule with a well-known WT appearance, the lobulated borders **(A)** or the cystic components **(C)**, as PA. The sonographic features demonstrate similarities of the lobulated borders (**A**, PA), (**B**, WT), and the cystic components (**C**, PA), (**D**, WT). The arrows indicate the lobulated borders of the masses, and the asterisks indicate the cystic components of the masses.

Ultrasound is the primary imaging modality for parotid gland neoplasm. However, the imaging features of PA and WT often overlap, making it challenging to establish a definitive distinction based solely on sonographic features. For instance, PA and WT patients commonly exhibit lobulated and cystic components in ultrasound images (14) (**Figures 1**, **2**). Consequently, relying solely on the subjective expertise of radiologists may compromise accuracy and efficiency (15, 16). FNAC is a widely accepted approach used for preoperative assessment in determining pathological natures of different tumors. However, for the differentiation of PA and WT, FNAC may yield non-diagnostic or intermediate results as FNAC can only observe cell types and morphology without comprehensively describing the tissue morphology or their relationship with surrounding tissues. Relying solely on FNAC is insufficient for accurately diagnosing PA and WT (17–19). Therefore, developing a reliable and objective discriminatory diagnostic method secondary to imaging and FNAC is necessary to improve the diagnostic accuracy for PA and WT.

Image-based deep learning (DL), as a potential solution, is emerging to enhance the diagnostic capabilities for physicians with promising results (15, 20). The utilization of DL models presents significant advantages over physician diagnosis in identifying challenging features, textures, and details, automatic feature learning, and effective depiction of complex structures (21). However, the DL algorithms used for parotid gland tumors were limited in the literature. Xia et al. successfully employed DL based on MRI images to classify salivary gland tumors with an accuracy of 82% in their 2021 study. However, their research primarily focused on differentiating between benign and malignant parotid gland tumors (22). To the best of our knowledge, there is a lack of research on utilizing DL approaches to differentiate PA from WT.

The objective of this study was to evaluate the feasibility of using DL models to distinguish PA and WT with the ultimate expectation that DL may furnish physicians with more dependable diagnostic evidence and provide surgeons with accurate treatment decisions and personalized clinical services.

## Methods

### Patients

With the ethical approval (No 050432-4-2018) of Fudan University Shanghai Cancer Center, we retrospectively enrolled 496 patients who underwent parotid gland surgery between January 2016 and December 2022, meeting the following inclusion criteria: (1) histopathological diagnosis of PA or WT; (2) ultrasonography examination of the parotid gland performed within one month prior to the surgical procedure. Eight patients were excluded for the following reasons: (1) simultaneous presence of both PA and WT in the parotid gland (n=3); (2) absence of corresponding two-dimensional ultrasonography image of the mass (n=5). Finally, a total of 488 cases were included. When allocating the research samples, randomization was conducted on the included patients to ensure the representativeness in model training and evaluation. The patients were randomly assigned in a ratio of 7:2:1, resulting in a training set comprising of 341 cases, a validation set consisting of 97 cases, and a testing set containing 50 cases.

Special attention was given to cases with inconclusive or erroneous FNAC results which were segregated into a distinct group, named as the indeterminate group. The performance of DL models for this group of patients was also evaluated. The disparity in case numbers between PA (96 cases) and WT (14 cases) within the indeterminate group necessitated a random selection of a specific number of cases from the remaining WT data (82 cases), aiming to rectify this imbalance. Finally, the total 192 patients were randomly divided into three sets - a training set (116 cases), a validation set (38 cases), and a testing set (38 cases) - with ratios of 6:2:2 respectively to ensure stability in subsequent DL models. Given the retrospective design, written informed consent was waived without disclosing identifiable information.

### Image acquisition and evaluation

US images of the PA and WT lesions were retrieved from the image archive database of Fudan University Shanghai Cancer Center. The latest US images of patients eligible for inclusion within one month before the surgery were collected from various ultrasound equipment models including GE V730 pro, Philips iU-22, Toshiba SSA-790, and Siemens S2000 with the linear array probe (7-14 MHz). All patients were supine during the examination, with their head and neck fully exposed. When examining one side, the head was slightly tilted to the opposite side, and after applying coupling agents, the probe lightly made contact with the skin. Continuous transverse, longitudinal, and oblique scans were performed on the parotid gland lesion area, and a multi-slice display was used to observe the lesion and preserve high-quality two-dimensional images. All images were downloaded in DICOM format at their original size and resolution.

Two experienced US physicians (Physicians A and B, with 5 and 10 years of clinical experience) independently identified PA and WT using ultrasound images. The two physicians were mutually blinded; neither knew previous radiological reports or patient clinical information. The diagnostic agreement of the two physicians was assessed using Cohen-Kappa coefficient. The performance of each physician was evaluated with the postoperative pathology result as the reference gold standard to determine the number of true positives, false positives, true negatives, and false negatives of the two physicians’ diagnoses. Based on these numbers, the sensitivity and specificity of each physician were calculated. The receiver operating characteristic (ROC) curve was plotted with sensitivity as the Y-axis and 1-specificity as the X-axis. Performance indicators, including the area under the ROC curve (AUROC), accuracy, sensitivity, specificity, positive predictive value (PPV), and negative predictive value (NPV), were used to evaluate the diagnostic performance of the physicians.

### Establishment of DL model and data preprocessing

Another two experienced US physicians who did not participate in the differentiation of PA and WT jointly selected the region of interest (ROI) from ultrasound images. One of the physicians outlined the ROI with the agreement of the other one. The ROI was a rectangular area embracing the tumor mass and approximately 5mm normal tissues surrounding the mass. The ROI selection was performed using the Labelme software developed by the Massachusetts Institute of Technology based on the Python framework (23). (https://sourceforge.net/projects/labelme/, accessed on 20 February 2023).

**Figure 3:**
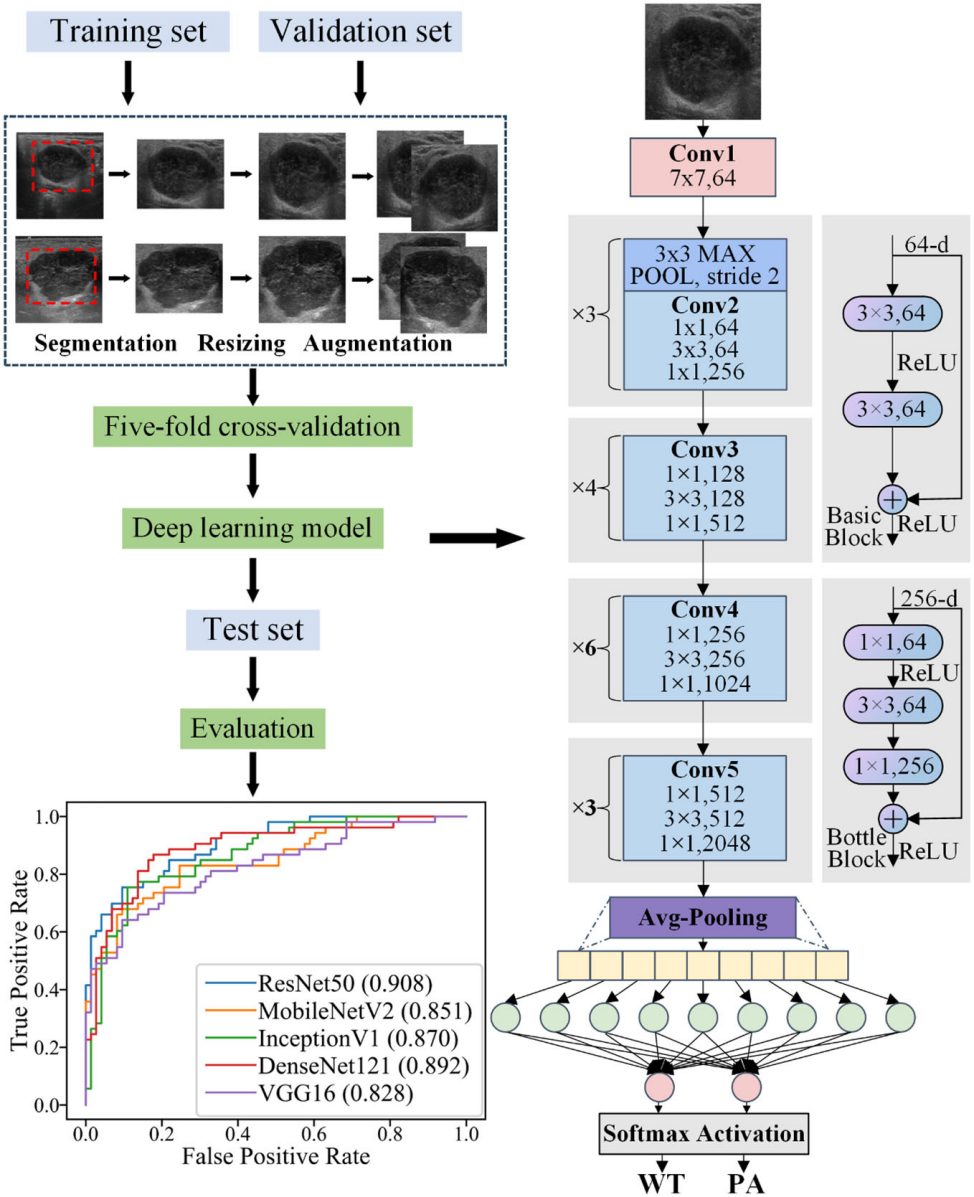
Schematic illustration of the workflow of using DL to differentiate parotid PA and WT.

The datasets underwent a standardized preprocessing workflow prior to establishing the five DL models (refer to **Figure 3**). For the training set, our preprocessing steps included resizing the image dimensions to 256 pixels. Subsequently, horizontal flips were applied on the images to enhance the generalization capability of the models. Finally, we transformed and normalized the processed images into tensors by setting each color channel’s mean and standard deviation to predefined values.

### DL model training

The DL model was established using Python as the programming language and PyTorch as the platform. Five network architectures, namely ResNet50, MobileNetV2, InceptionV1, DenseNet121, and VGG16, were employed for image classification tasks. The pre-trained models of these networks were utilized. To mitigate overfitting risks, a five-fold cross-validation was implemented. For each fold, the model underwent training on the training set and evaluation on the validation set. Following each training and evaluation phase, the AUROC of the current fold in the validation set was calculated. Once all folds had been trained and evaluated, the average AUROCs of all folds were computed and plotted accordingly. Finally, to assess performance on the indeterminate group, the model was retrained using both training and validation data combined before being evaluated on the test set with a ROC being plotted.

### Assessment of DL models

The aim of this study was to develop a binary classification model capable of effectively distinguishing PA and WT. Postoperative pathology was used as the reference gold standard. The performance of DL models were evaluated in terms of AUROC, accuracy, sensitivity, specificity, PPV, NPV and Mathew’s Correlation Coefficient (MCC) for both the overall group and the indeterminate group.

### DL model interpretation

In tumor classification tasks, it is crucial to identify the ROI in the image that the model primarily focuses on. This elucidates the decision-making process of the DL model. Therefore, we employed Gradient-weighted Class Activation Mapping (Grad-CAM), a visualization technique used to highlight the ROI in an image, to ensure that our DL model was based on the mass area (using ResNet50 as an example). For this specific classification task, we selected ResNet50’s final convolutional layer which typically encapsulates ample information and captures complex features most effectively. The generated heatmap is a two-dimensional tensor where each element represents its corresponding pixel’s contribution to predicting outcomes. The “hot” regions in the heatmap correspond to the areas used for outcome prediction by the DL model.

The heatmap was eventually superimposed onto the original image, resulting in a composite that effectively showcases the ROI. This visualization technique enables accurate identification of tumor boundaries and internal structures for classification purposes. Moreover, the transparency inherent in this process enhances the reliability of the model while providing valuable feedback for further improvement and validation.

### Statistical analysis

The clinical characteristics of all patients, including age, gender, and maximum diameter of the tumor mass in the ultrasound, were categorized based on the training set, validation set, and test set. Quantitative variables were described using mean ± standard deviation (Mean ± SD), while categorical variables were presented as frequency distributions. Patients were stratified into two ordinal levels according to age (≤50y and >50y) and maximum diameter (≤20mm and >20mm). Chi-square test was utilized to compare these categorical data among the three groups. Statistical analysis was conducted using SPSS version 25.0 (SPSS Inc., Chicago, IL, USA). A two-tailed P value less than 0.05 was considered statistically significant.

## Results

### Patient characteristics

The study enrolled a total of 488 patients, with 266 (54.5%) diagnosed with PA and 222 (45.5%) with WT. The age range of the participants was between 14 and 82 years, with an average age of 52 years (SD: 13.8). Among them, there were 183 females and 305 males. The mean maximum diameter measured by ultrasound was 27.1 mm (SD:10.2). No statistically significant differences were observed in terms of age, gender, and maximum diameter among the three datasets in the two groups (P>0.05) (**Tables 1**, **2**).

**Table 1:** Comparison of clinical features between three datasets in all cases.

	Training set (n=341)	Validation set (n=97)	Test set (n=50)	P value
**Age**				0.078
≤50	149	30	20	
>50	192	67	30	
**Gender**				0.864
Male	212	60	33	
Female	129	37	17	
**Maximum diameter**				0.374
≤20	94	20	14	
>20	247	77	36	

**Table 2:** Comparison of clinical features between three datasets in the indeterminate group.

	Training set (n=116)	Validation set (n=38)	Test set (n=38)	P value
**Age**				0.923
≤50	43	13	13	
>50	73	25	25	
**Gender**				0.073
Male	80	20	29	
Female	36	18	9	
**Maximum diameter**				0.293
≤20	33	13	7	
>20	83	25	31	

**Table 3** shows the clinical characteristics and pathological indicators of patients: 1) Malignant transformation occurred in only 7 cases (2.7%) of PA patients and 1 case (0.5%) of WT patients. 2) Atypical hyperplasia was exhibited by 19 (7.1%) PA patients, while only 1 (0.5%) WT patient presented with atypical hyperplasia. 3) Capsule invasion was observed in 21 cases (7.9%) of PA patients, whereas no WT patient showed evidence of capsule invasion.

**Table 3:** Patient and tumor characteristics.

	PA (n=266)	WT (n=222)	%
Age			
≤50	177	20	40.4
>50	89	202	59.6
Gender			
Male	93	212	62.5
Female	173	10	37.5
Maximum diameter			
≤20	102	26	26.2
>20	164	196	73.8
Malignant transformation			
yes	7	1	1.6
no	259	221	98.4
Atypical hyperplasia			
yes	19	1	4.1
no	247	221	95.9
Invasion of the capsule			
yes	21	0	4.3
no	245	222	95.7

### Performance of US physicians and FNAC

The diagnostic performance of the US physicians and FNAC is presented in **Table 4**. The average AUROC and accuracy for the two US physicians were merely 0.475 and 0.576, indicating a limited discriminatory ability. The Cohen-Kappa coefficients between the two US physicians was 0.457 (p<0.05). FNAC demonstrated an AUROC of 0.721, showcasing high specificity (0.932) but relatively lower sensitivity (0.510).

**Table 4:** The diagnostic performance of two physicians and FNAC.

	AUROC	Accuracy	Sensitivity	Specificity	PPV	NPV
**Physician A**	0.351	0.545	0.320	0.617	0.569	0.500
**Physician B**	0.598	0.606	0.695	0.500	0.625	0.578
**FNAC**	0.721	0.699	0.510	0.932	1.000	1.000

FNAC, Fine Needle Aspiration cytology; AUROC, Area Under the Receiver Operating Characteristic; PPV, Positive Predictive Value; NPV, Negative Predictive Value.

### Performance of DL models

#### Differentiation of PA and WT in all cases

**Figure 4:**
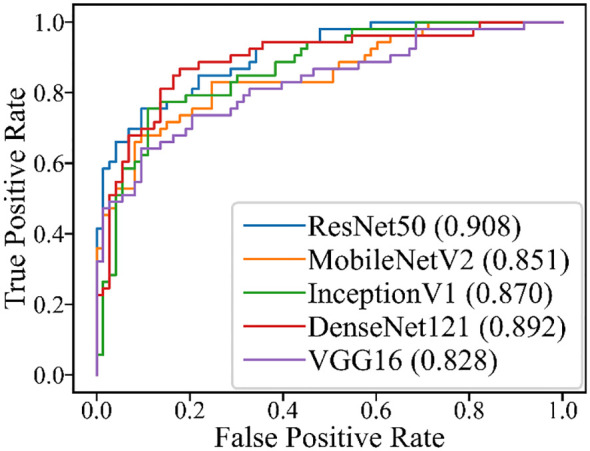
ROC for the five DL models in the test set of all cases.

The performance of the five DL models in distinguishing PA and WT on the test set is presented in **Table 5** and **Figure 4**. Among these models, ResNet50 achieved the highest AUROC of 0.908, followed by DenseNet121 with an AUROC of 0.892, InceptionV1 with an AUROC of 0.870, MobileNetV2 with an AUROC of 0.851, and VGG16 with an AUROC of 0.828. Additionally, detailed evaluation metrics including accuracy, sensitivity, specificity, PPV, and NPV are provided for each model as shown in **Table 5**.

**Table 5:** Performance evaluation of DL models in differentiating PA and WT for all cases.

Models	AUROC	Accuracy	Sensitivity	Specificity	PPV	NPV	MCC
**ResNet50**	0.908	0.833	0.736	0.904	0.848	0.825	0.656
**MobileNetV2**	0.851	0.778	0.830	0.740	0.698	0.857	0.563
**InceptionV1**	0.870	0.762	0.849	0.700	0.672	0.864	0.542
**DenseNet121**	0.892	0.802	0.698	0.877	0.804	0.800	0.589
**VGG16**	0.828	0.722	0.792	0.671	0.636	0.817	0.458

PA, pleomorphic adenoma; WT, Warthin tumor; AUROC, Area Under the Receiver Operating Characteristic; PPV, Positive Predictive Value; NPV, Negative Predictive Value; MCC, Mathew’s Correlation Coefficient.

#### Differentiation of PA and WT in the indeterminate group

**Figure 5:**
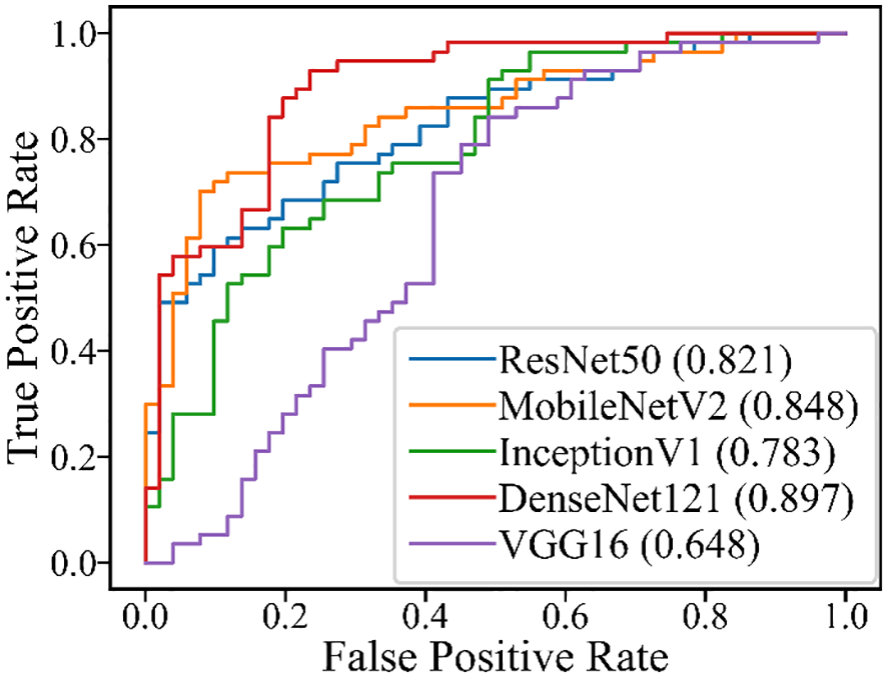
ROC for the five DL models in the test set of the inderterminate group.

**Table 6** and **Figure 5** show the performance of the five DL models in distinguishing PA and WT for the indeterminate group. DenseNet121 model exhibited the optimal performance: AUROC (0.897), accuracy (0.806), sensitivity (0.789), specificity (0.824), PPV (0.833), NPV (0.778), and MCC (0.612). Conversely, the VGG16 model had the worst performance: AUROC (0.648), accuracy (0.676), sensitivity (0.825), specificity (0.510), PPV (0.653), NPV (0.722), and MCC (0.354).

**Table 6:** Performance evaluation of DL models in differentiating PA and WT for the indeterminate group.

Models	AUROC	Accuracy	Sensitivity	Specificity	PPV	NPV	MCC
**ResNet50**	0.821	0.722	0.701	0.745	0.755	0.691	0.446
**MobileNetV2**	0.848	0.750	0.842	0.647	0.727	0.786	0.501
**InceptionV1**	0.783	0.713	0.684	0.745	0.750	0.679	0.429
**DenseNet121**	0.897	0.806	0.789	0.824	0.833	0.778	0.612
**VGG16**	0.648	0.676	0.825	0.510	0.653	0.722	0.354

PA, pleomorphic adenoma; WT, Warthin tumor; AUROC, Area Under the Receiver Operating Characteristic; PPV, Positive Predictive Value; NPV, Negative Predictive Value; MCC, Mathew’s Correlation Coefficient.

#### Robustness and visualization of the DL models

**Figure 6:**
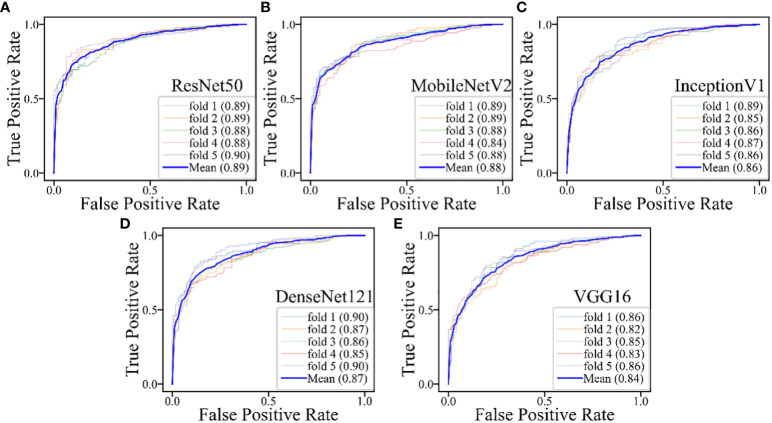
The performances of five-fold cross-validation in all DL models for all cases: **(A)** the ResNet50 model; **(B)** the MobileNetV2 model; **(C)** the InceptionV1 model; **(D)** the DenseNet121 model; **(E)** the VGG16 model.

**Figure 7:**
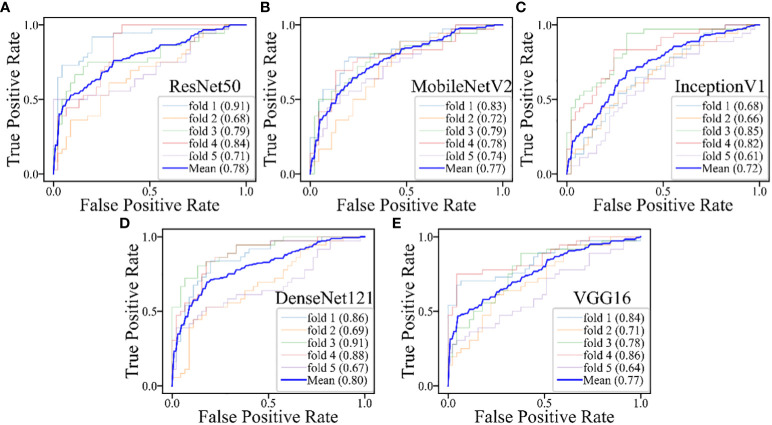
The performances of five-fold cross-validation in DL models for the inderteminate group: **(A)** the ResNet50 model; **(B)** the MobileNetV2 model; **(C)** the InceptionV1 model; **(D)** the DenseNet121 model; **(E)** the VGG16 model.

**Figure 8:**
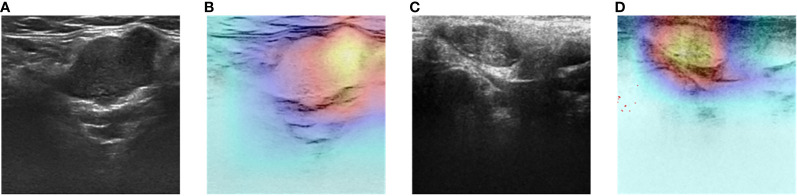
Two-dimensional ultrasound images and heatmaps of parotid gland tumor: **(A)** ultrasound image of PA; **(B)** heatmap of the same PA; **(C)** ultrasound image of WT; **(D)** heatmap of the same WT.

The DL models demonstrated consistent performances in the five-fold cross-validation results for distinguishing between PA and WT, with AUROC values remaining stable across different models, indicating no evidence of overfitting (**Figure 6**). This further validates the robustness of the DL models. Similarly, in the five-fold cross-validation results for the indeterminate group, the DL models also exhibited similar performances without any indications of overfitting (**Figure 7**). Heatmaps generated using the RestNet50 model revealed that the ROI used in the DL model was primarily within the mass area and the junction with parotid tissue, while peripheral normal parotid tissue had a minor impact (**Figure 8**).

## Discussion

In this study, we evaluated five DL models based on US images to differentiate PA and WT. The five DL models presented comparable good performances. Among these models, ResNet50 achieved the highest AUROC, followed by DenseNet121, InceptionV1, MobileNetV2, and VGG16 (0.828-0.908). These performances of all DL models were superior to US physicians and FNAC. For the indeterminate group that was inconclusive by FNAC, DL models also provide acceptable results (AUROC:0.648-0.897).

PA is the most common salivary gland tumor, accounting for 60% of these neoplasms. If untreated, 6.2% of PA may undergo malignant transformation to CXPA (24). The incomplete resection of the PA tumor mass may increase the risk of its transformation to CXPA and recurrence (25). It was reported that the recurrence rate ranges between 20%-45% following tumor enucleation and 3.4%-6.8% after partial or complete superficial parotidectomy (26–30). The increased recurrence rate may be attributed to the tumor’s growth pattern, which progressively expands and infiltrates the surrounding tissue, ultimately breaching its encapsulation and giving rise to satellite nodules. Moreover, there is an elevated risk for malignant transformation in PA with the prolonged observation time. One observational research suggests that the incidence rate for malignant transformation in PA is 1.5% at 5 years and rises to 9.5% at 15 years after the surgery (9). On the contrary, WT presented lower recurrence rate. A study conducted at Johns Hopkins University reported a lower recurrence rate (4.2%) and malignant transformation rate (1%) in WT (31). Some researchers have even hypothesized that WT may not represent a genuine neoplasm but rather an inflammatory or delayed hypersensitivity reaction (32).

Our results reinforced the biological and behavioral disparities between PA and WT. We found that 7 PA cases (2.7%) experienced malignant transformations compared to only 1 WT case (0.5%). This indicated that PA carries a higher risk for malignant transformation than WT, which accords with the behavior of CXPA. Meanwhile, atypical hyperplasia was observed in 19 PA cases (7.1%) and 1 WT case (0.5%). Involvement of capsule occurred in 21 PA cases (7.9%), while no instances were observed among PT cases. This emphasizes the infiltrative nature of PA towards adjacent structures, which sharply contrasts with the benign characteristics of WT. The variations in malignant transformation, atypical hyperplasia, and capsule invasion underscore the significance of accurately diagnosing PA and WT.

Ultrasound is the preferred modality for distinguishing between PA and WT due to its advantages of high resolution, non-ionizing radiation, and cost-effectiveness. However, accurately differentiating these two tumors based on sonographic characteristics such as multifocality, morphology, internal cystic changes, and tumor vascularity remains a challenging task (12, 14, 33). Our study revealed that the average diagnostic accuracy of the two US physicians was only 57.6%, which aligns with previous research findings (34, 35). Meanwhile, the diagnostic agreement between the two physicians was low with the Cohen-Kappa coefficient of 0.457. The low diagnostic agreement and accuracy of US physicians verified the limitation of differentiating PA and WT by naked eyes.

In this study, FNAC demonstrated a diagnostic accuracy of only 0.699. The sensitivity was 0.510, while specificity was determined to be 0.932; PPV and NPV were reported as equal to one, respectively. These results indicate that FNAC has a high rate of false-negative diagnoses but a low rate of false-positive diagnoses in identifying PA and WT in this particular study which was consistent with existing literature reports (17, 19). This can be explained by the fact that an accurate diagnosis of PA and WT relies on evaluating cell arrangement and growth patterns which are not adequately represented in FNAC samples. Moreover, the absence of clear guidelines for reporting and interpreting FNAC results poses challenges for pathologists in making definitive diagnoses (17–19).

DL, as an important branch of artificial intelligence algorithms, was initially proposed by Hinton in 2006. It involves training a multi-layered deep network structure using specific samples. DL models have the capability to detect unique textures that may pose challenges for human visual perception and accurately quantify image features, thereby reducing subjective interpretation (21, 36). In comparison to traditional machine learning methods reliant on feature extraction, DL offers several advantages. Firstly, it automatically amalgamates low-level features to form high-level abstract features, eliminating human subjectivity. Secondly, DL models eliminate the need for imaging physicians to precisely delineate tumor margins, thus minimizing inter-operator differences in identifying regions of interest (ROI). Thirdly, DL models effectively integrate tumor image information with clinical data, providing a more precise foundation for clinical decision-making (37).

Each of the five DL models utilized in our study possesses distinct characteristics. ResNet50 employs residual connections to address the issue of vanishing and exploding gradients, utilizing “residual learning” (38). MobileNetV2 is a lightweight network suitable for mobile and embedded devices, employing Depthwise Separable Convolution to reduce complexity and size (39). InceptionV1 incorporates “Inception modules” with parallel convolutional kernels to capture features at various scales (40). DenseNet121 enhances gradient propagation and feature reuse while reducing parameters by connecting each layer to all previous layers through feature map concatenation (41). VGG16 follows a regular structure that utilizes al kernels and 2×2 maximum pooling layers (42).

This is the first study in the literature that using ultrasound images-based DL algorithms to differentiate PA and WT. The comparable performances exhibited by the five DL models accurately distinguish PA from WT lesions while precisely identifying the tumor mass as the ROI, thereby rendering our results robust and promising. In the future, more related studies are warranted to reinforce our findings. The potential of ResNet50 model as the best DL model for differentiating PA and WT at US is also warranted for further validation.

There are several limitations in our study. Firstly, the data were retrospectively collected from various US instruments and physicians, which may introduce variability. Secondly, due to different evaluation methods, we did not statistically compare the performance between physicians and DL models. Thirdly, our study only focused on PA and WT; therefore, this model may not be applicable to all benign parotid tumors. Nevertheless, these two types of tumors account for approximately 93% of such cases. Finally, since all our data came from a single center without external validation, we plan to collaborate with multiple centers to address these limitations and enhance the practicality as well as the generalizability of DL models.

## Conclusion

DL models based on ultrasound images can differentiate PA and WT in parotid gland tumors with reliable results. They provided a feasible supplementary approach for preoperative diagnosis of PA and WT secondary to imaging and FNAC. This will thereafter provide valuable information for surgeons to select appropriate surgical interventions. Future multicenter studies are expected to yield more robust results.

## Data availability statement

The raw data supporting the conclusions of this article will be made available by the authors, without undue reservation.

## Ethics statement

The studies involving humans were approved by The Ethics Committee of Fudan University Shanghai Cancer Center. The studies were conducted in accordance with the local legislation and institutional requirements. The ethics committee/institutional review board waived the requirement of written informed consent for participation from the participants or the participants’ legal guardians/next of kin because It’s not involved in this retrospective study. Written informed consent was not obtained from the individual(s) for the publication of any potentially identifiable images or data included in this article because it's not involved in this retrospective study.

## Author contributions

XL: Methodology, Writing – original draft. YM: Software, Writing – review & editing. LQ: Data curation, Writing – review & editing. ZS: Conceptualization, Writing – review & editing. YW: Formal Analysis, Writing – review & editing. JS: Supervision, Writing – review & editing. CC: Supervision, Writing – review & editing. JC: Investigation, Writing – review & editing. JC: Validation, Writing – review & editing. JL: Supervision, Writing – review & editing.
